# Increased joint line obliquity is associated with lateral cartilage degeneration after medial opening wedge high tibial osteotomy: a quantitative MRI analysis

**DOI:** 10.1186/s43019-026-00322-8

**Published:** 2026-06-04

**Authors:** Takaaki Hiranaka, Giacomo Dal Fabbro, Kieran O’Brien, James Linklater, David A Parker

**Affiliations:** 1 Sydney Orthopaedic Research Institute, Sydney, Australia; 2 Department of Sports Medicine, Faculty of Medicine, Dentistry and Pharmaceutical Sciences, Okayama University, Okayama, Japan; 3 Siemens Healthineer, Brisbane, Australia; 4 Department of Musculoskeletal Imaging, Castlereagh Imaging, Sydney, Australia

## Abstract

**Background:**

Joint line obliquity (JLO) often increases after medial opening wedge high tibial osteotomy (MOWHTO), and excessive JLO may adversely affect the lateral compartment. However, its effects on cartilage tissue and postoperative outcomes remain unclear. This study investigated whether increased postoperative JLO is associated with early cartilage changes on quantitative MRI *T*2 mapping and differences in clinical outcomes.

**Methods:**

A total of 33 knees undergoing MOWHTO were retrospectively reviewed. Radiographic parameters—hip-knee-ankle angle (HKA), medial proximal tibial angle (MPTA), and JLO—were assessed preoperatively and at 6 months. Based on postoperative radiographic measurements, patients were classified as increased JLO/MPTA (Group I: JLO > 5° and/or MPTA > 94°) or acceptable range JLO/MPTA (Group A: JLO < 5° and MPTA < 94°). Quantitative MRI *T*2 mapping was performed preoperatively and at 12 months to measure *T*2 relaxation times (*T*2 values) and Δ*T*2 values (postoperative–preoperative) in the patella, trochlea, lateral femoral condyle (LFC), and lateral tibial plateau (LTP), analyzing superficial, deep, and total cartilage layers. Knee Injury and Osteoarthritis Outcome Score (KOOS) was assessed at both time points.

**Results:**

Group I included 13 patients and group A included 20 patients. Postoperative HKA was similar between groups (*p* = 0.326). No significant intergroup differences were observed in preoperative or postoperative *T*2 values in the patellofemoral or lateral compartments. However, Δ*T*2 values in the LTP were significantly higher in group I than in group A in the superficial (3.3 ± 6.5 versus −1.7 ± 5.9 ms, *p* = 0.022) and total (4.5 ± 5.7 versus − 0.6 ± 5.0 ms, *p* = 0.012) layers. Group I also had worse KOOS–Symptoms (*p* = 0.016), QOL (*p* = 0.022), and Sport/Rec (*p* = 0.040).

**Conclusions:**

Even with similar postoperative limb alignment, increased JLO/MPTA was associated with early degenerative changes in lateral tibial cartilage and worse clinical outcomes. Maintaining postoperative JLO within the acceptable range during MOWHTO may help maintain lateral compartment cartilage health and optimize clinical outcomes.

*Level of Evidence*: Level IV.

## Background

Medial opening wedge high tibial osteotomy (MOWHTO) is a widely accepted joint-preserving surgical option for active patients with medial compartment osteoarthritis (OA) and varus malalignment [1, 2]. By shifting the mechanical axis laterally, the procedure reduces loading on the medial compartment, alleviates pain, and can delay the need for knee arthroplasty [3, 4]. Because MOWHTO corrects alignment solely through tibial osteotomy, it inherently increases the medial proximal tibial angle (MPTA) and may also increase joint line obliquity (JLO). To achieve the target valgus alignment, this approach can sometimes result in excessive MPTA or JLO [5, 6].

Several clinical studies have reported that greater postoperative JLO is associated with inferior patient-reported outcomes, lateral compartment pain, and medial joint space narrowing [7–11]. Biomechanically, such alterations have been linked to changes in load distribution, with increased load in the lateral compartment [12], and excessive shear stress in the tibial articular cartilage [13], potentially accelerating degenerative processes. Conversely, a number of high-quality studies have found that increased JLO does not significantly affect postoperative clinical outcomes, cartilage status, or long-term survivorship, making the clinical relevance controversial [8, 14–16]. This inconsistency may be largely attributable to heterogeneity in JLO measurement methods and cut-off definitions, as well as inadequate control of key confounding factors, underscoring the need for further biomechanically grounded investigations.

Although multiple studies have examined the relationship between JLO and clinical outcomes after MOWHTO, most have relied primarily on patient-reported outcomes and radiographic assessments. Radiographic assessment is particularly limited in detecting changes in the short to intermediate term. *T*2 mapping, a quantitative magnetic resonance imaging (MRI) technique, addresses this limitation by detecting biochemical changes in cartilage—such as alterations in collagen fiber orientation and water content—allowing early degeneration to be identified while morphology remains intact [17–19]. Prolonged *T*2 relaxation times (*T*2 values) indicate cartilage degeneration or inflammation, reflecting increased water content, reduced proteoglycans, and loss of collagen structure [20]. Therefore, *T*2 mapping provides an appropriate modality to investigate the potential impact of postoperative JLO on cartilage status following MOWHTO, without the need to wait for longer term radiographic changes.

To date, only a few studies have investigated the effect of postoperative JLO on cartilage after MOWHTO, and none have used quantitative techniques such as *T*2 mapping. Therefore, the purpose of this study was to investigate whether increased postoperative JLO/MPTA is associated with early cartilage changes in the lateral and patellofemoral compartments detectable by quantitative MRI *T*2 mapping, and to compare patient-reported clinical outcomes between groups. We hypothesized that patients with greater postoperative JLO and/or MPTA would exhibit increased *T*2 values in these compartments and worse patient-reported outcomes.

## Methods

### Patient selection

A retrospective analysis was performed on a consecutive series of patients with symptomatic medial compartment knee osteoarthritis and varus malalignment who underwent MOWHTO between April 2014 and August 2023. Data were collected prospectively as part of routine clinical practice at our institution. Inclusion criteria were: (1) medial compartment knee osteoarthritis with varus malalignment unresponsive to conservative treatment and (2) availability of preoperative and postoperative full-length standing radiographs, *T*2 mapping scans, and clinical outcome scores. Exclusion criteria were: (1) procedures other than MOWHTO, such as medial closed wedge distal femoral osteotomy or double-level osteotomy; (2) radiographs or MRI scans of insufficient quality for accurate measurement; or (3) missing imaging or clinical outcome data. This study was approved by the Human Research Ethics Committee of the Northern Sydney Local Health District (Reference: HREC/17/HAWKE/140), and written informed consent was obtained from all participants.

### Preoperative planning and surgical technique

All procedures were performed by experienced knee surgeons using a standardized MOWHTO technique. Osteotomy execution was guided by either a patient-specific instrument or an image-free navigation system to improve accuracy. Fixation was achieved with a locking plate specifically designed for MOWHTO. The target weight-bearing line ratio was set between 55 and 62.5% on the basis of the degree of osteoarthritis and concomitant procedures. Postoperative rehabilitation followed a structured protocol, beginning with 4 weeks of protected weight-bearing and progressing to full weight-bearing by 8 weeks, based on clinical and radiographic evaluations.

### Image acquisition

Preoperative standing long-leg EOS radiographs were obtained in double-leg stance for the coronal plane and in single-leg stance for the sagittal plane (EOS imaging Inc., Paris, France). High-resolution MRI was performed using a 3.0-T scanner (Magnetom Skyra; Siemens Healthineers, Erlangen, Germany) with a 15-channel phased-array knee coil. Sagittal multi-echo spin-echo (MESE) sequences for quantitative *T*2 mapping were acquired preoperatively and at 12 months postoperatively, with the knee fixed at 15° of flexion and maintained in neutral rotation to ensure reproducibility and minimize motion artifacts. Acquisition parameters were standardized across all scans as follows: repetition time (TR) = 2150 ms, echo times (TEs) = 13.8, 27.6, 41.4, 55.2, and 69 ms; slice thickness = 3 mm; field of view (FOV) = 160 × 160 mm; acquisition matrix = 326 × 384; bandwidth = 228 Hz/pixel; and total acquisition time (TA) = 7 min 22 s. Quantitative *T*2 maps were generated inline on the scanner using the vendor’s mono-exponential fitting algorithm (MapIt, Siemens Healthineers) applied on a pixel-by-pixel basis with default settings.

### Radiographic evaluation

**Fig. 1:**
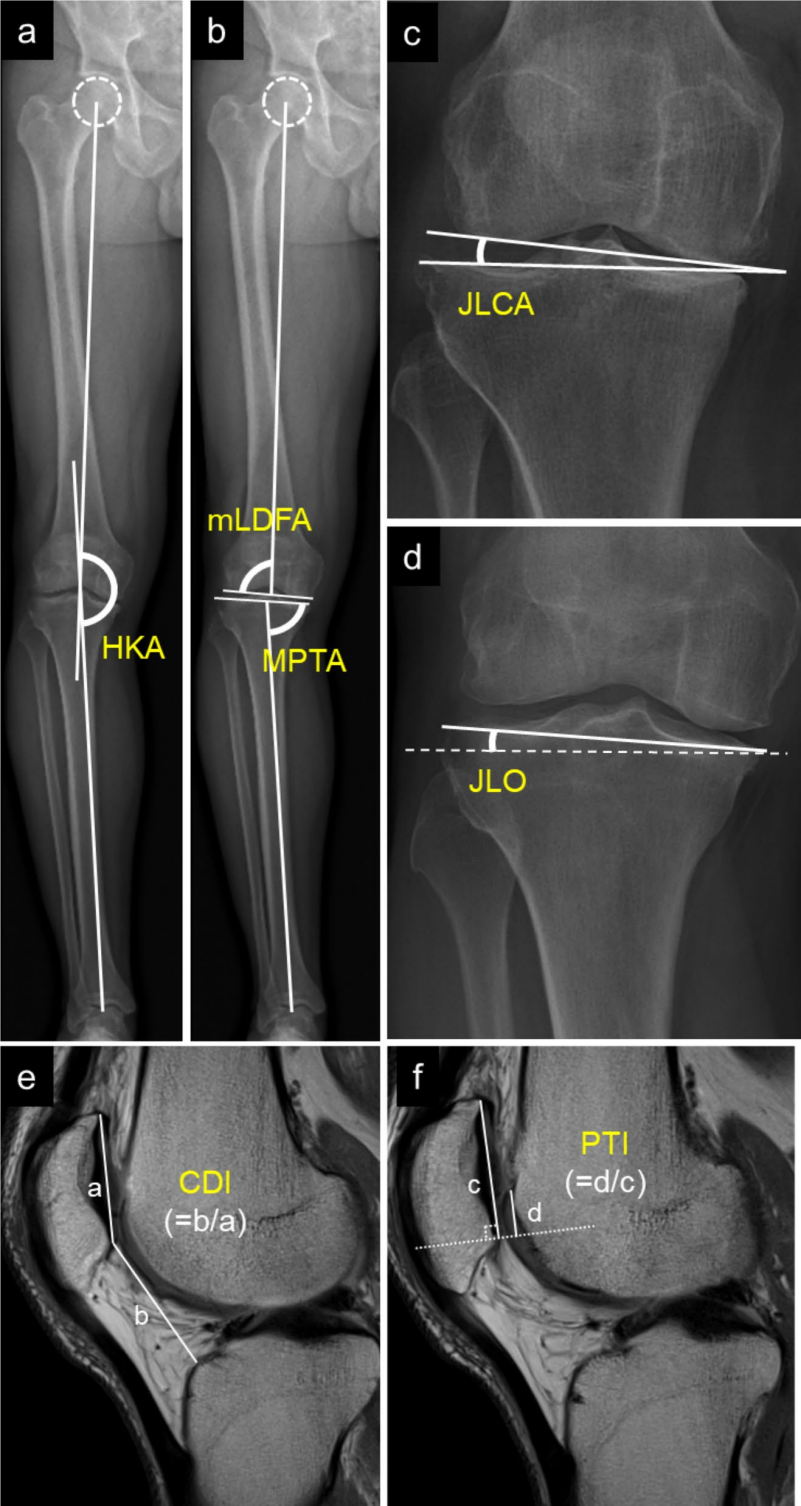
Imaging-based measurements, including standing long-leg EOS radiographs (**A**–**D**) and sagittal *T*2 mapping sequence magnetic resonance imaging (MRI) (**E**, **F**). **A**, **B** EOS radiographs illustrating the measurement of HKA, MPTA, and mLDFA. **C** Measurement of JLCA. **D** Measurement of JLO, defined as the angle between a line parallel to the ground and the tangential line of the tibial plateau, with valgus recorded as positive and varus as negative. **E** MRI measurement of the CDI, calculated as the ratio of the distance from the lower pole of the patellar articular surface to the tibial plateau (b) to the length of the patellar articular surface (a). **F** MRI measurement of the PTI, defined as the ratio of the length of trochlear cartilage overlapping the patella (d) to the patellar articular cartilage length (c)

Radiographic assessments were conducted preoperatively and at 6 months postoperatively using EOS standing long-leg radiographs. Each radiograph included the hip, knee, and ankle joints in a single image. Prior to image acquisition, limb rotation was adjusted by the radiology technician to obtain a true anteroposterior view, with the patella centered over the femoral condyles to minimize the effect of rotational malalignment. The evaluated parameters included hip-knee-ankle angle (HKA), MPTA, mechanical lateral distal femoral angle (mLDFA), joint line convergence angle (JLCA), and JLO. The JLO was defined as the angle between a line parallel to the ground and the tangential line of the tibial plateau, with valgus joint line recorded as positive and varus joint line as negative (Fig. 1A–D).

Patellar height was assessed using sagittal images acquired from the *T*2 mapping sequence preoperatively and at 12 months postoperatively. Measurements included the Caton–Deschamps index (CDI) and patellotrochlear index (PTI). Measurements were performed on the mid-sagittal slice of the patella that demonstrated the greatest patellar length and the thickest articular cartilage. CDI was calculated as the ratio between the distance from the lower pole of the patellar articular surface to the tibial plateau and the length of the patellar articular surface [21]. A CDI value between 0.8 and 1.2 is generally considered normal. PTI was defined as the ratio of the length of trochlear cartilage overlapping the patella to the patellar articular cartilage length, with values between 0.125 and 0.50 regarded as normal [22] (Fig. 1E, F).

Patients were classified into two groups according to postoperative JLO and MPTA, following the recommendations of the recent ESSKA international consensus [23]. The increased JLO/MPTA group (Group I; JLO > 5° and/or MPTA > 94°) exceeded the suggested threshold for acceptable coronal alignment, whereas the acceptable range JLO/MPTA group (Group A; JLO < 5° and MPTA < 94°) was within the recommended range based on postoperative radiographic measurements.

### Region of interest (ROI) placement

**Fig. 2:**
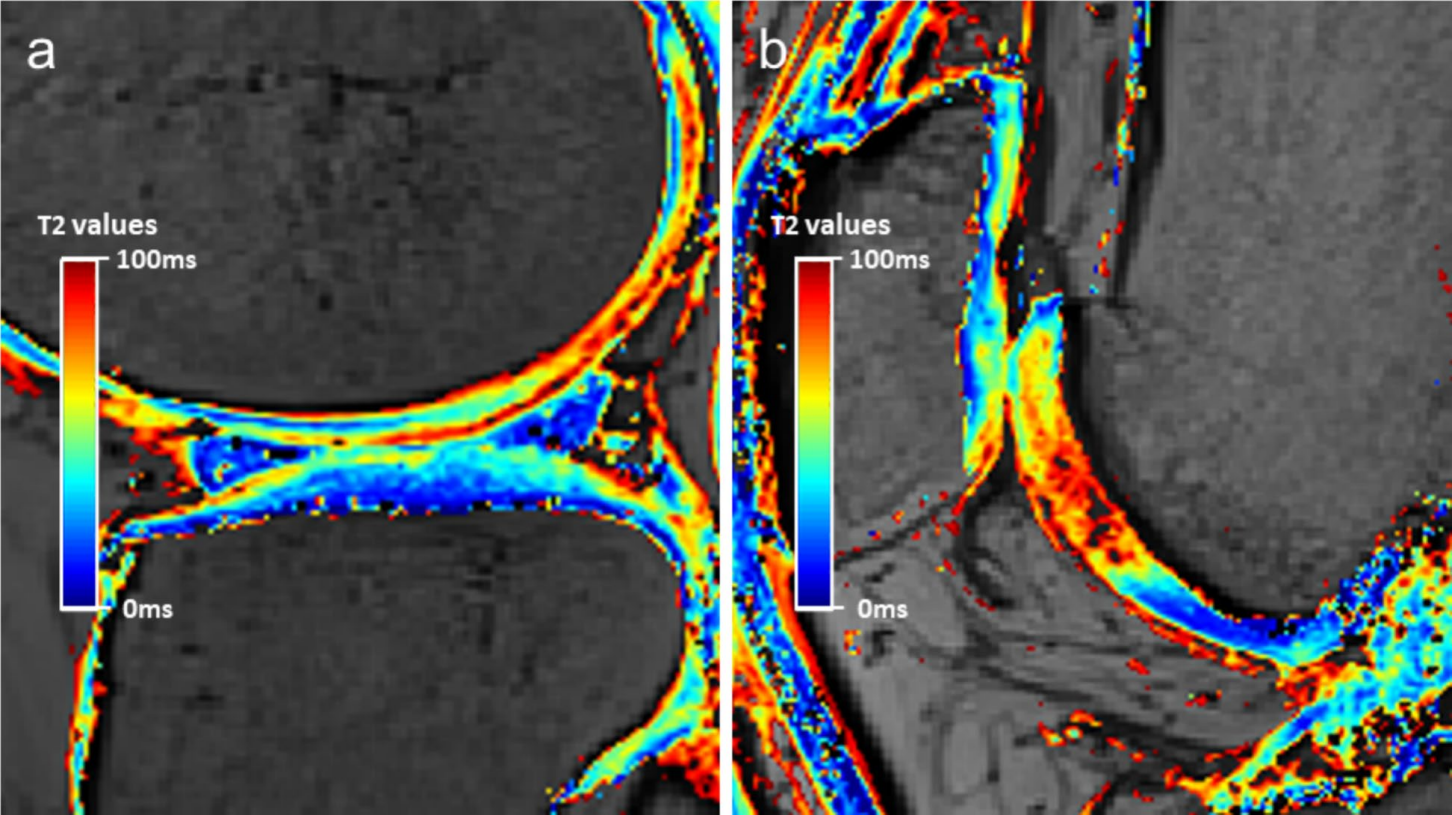
Representative sagittal *T*2 mapping images illustrating the ROI placement for cartilage segmentation. **A** Lateral compartment. **B** PF compartment. Color scale indicates *T*2 values in milliseconds. The color scale indicates *T*2 values in milliseconds, with lower *T*2 values (blue) representing shorter relaxation times and higher *T*2 values (red) representing longer relaxation times, which are associated with cartilage matrix changes or degeneration

**Fig. 3:**
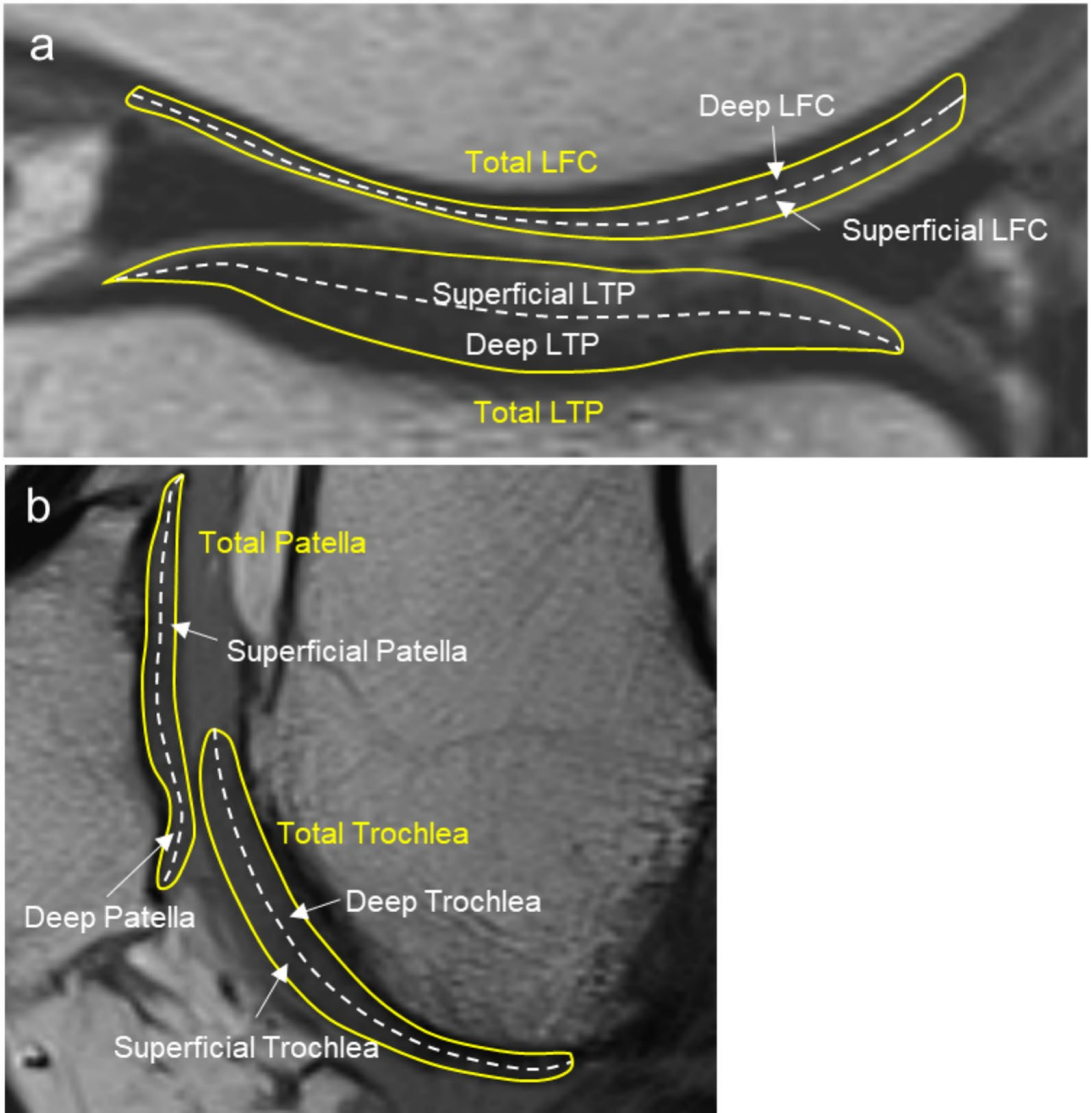
Representative sagittal images demonstrating the division of each ROI into superficial, deep, and total cartilage layers for quantitative *T*2 analysis. **A** Lateral compartment: LFC and LTP. **B** PF compartment: patella and trochlea. Solid yellow lines outline the total cartilage, and dashed white lines indicate the boundary between superficial and deep layers

Segmentation of the articular cartilage was performed for two compartments: the patellofemoral (PF) compartment (patella and trochlea) and the lateral compartment (lateral femoral condyle [LFC] and lateral tibial plateau [LTP]). *T*2 mapping images were acquired preoperatively and at 12 months postoperatively. In the lateral compartment, ROIs were placed on the sagittal slice passing through the midpoint of the medial–lateral axis of the LTP to evaluate the articular cartilage of the LFC and LTP, while in the PF compartment they were placed on the sagittal slice where the patella demonstrated its greatest proximal–distal dimension to evaluate the articular cartilage of the patella and trochlea [24–26] (Fig. 2). For each compartment, a single sagittal slice was selected. The cartilage was manually delineated to exclude subchondral bone and synovial fluid, and further divided into superficial and deep layers of equal thickness to evaluate zonal variation. A full-thickness (total) layer was also analyzed. The mean *T*2 value of the selected slice was defined as the *T*2 value for each ROI in the superficial, deep, and total layers (Fig. 3). All manual segmentations were performed using ImageJ software (National Institutes of Health, Bethesda, MD, USA) by two fellowship-trained orthopedic surgeons, and independently reviewed and verified by a musculoskeletal radiologist with over 20 years of experience.

### Patient reported outcome measures (PROMs)

PROMs were assessed in person at the time of the initial surgery and again at 12 months postoperatively using the Knee Injury and Osteoarthritis Outcome Score (KOOS) [27] and the Western Ontario and McMaster Universities Arthritis Index (WOMAC) [28]. The KOOS evaluates five domains: pain, symptoms, daily living activities (ADL), sports and recreational activities (Sport/Rec), and knee-related quality of life (QOL) [27]. The WOMAC is composed of three sections that assess pain (5 items), stiffness (2 items), and functional ability (17 items). Each item is scored on a scale of 0–4, resulting in maximum scores of 20 for pain, 8 for stiffness, and 68 for function. The overall WOMAC score ranges from 0 to 96, with higher scores indicating greater severity of symptoms [28]. The improvements in KOOS (ΔKOOS) and WOMAC (ΔWOMAC) were calculated as the postoperative scores minus the preoperative scores.

### Statistical analysis

All statistical analyses were performed using IBM SPSS Statistics for Windows, version 29.0 (IBM Corp., Armonk, NY, USA). Between-group comparisons of demographic, radiographic, *T*2 mapping, and PROM variables were performed using the independent-samples *t*-test. Categorical variables were compared using the chi-square test. Sensitivity analyses were performed to assess the robustness of the primary findings by adjusting for baseline alignment differences (HKA, mLDFA, and JLO) using a general linear model with covariates. A *p*-value < 0.05 was considered statistically significant. Interobserver and intraobserver reliabilities were assessed using the intraclass correlation coefficient (ICC), with values > 0.75 indicating satisfactory reliability. For interobserver reproducibility, two experienced orthopedic surgeons independently evaluated 10 randomly selected *T*2 mapping scans. Intraobserver repeatability was assessed by having the same observer re-evaluate the same scans after a 2-week interval.

## Results

### Inter-observer reproducibility and intra-observer repeatability

Quantitative MRI *T*2 mapping measurements in the lateral compartment demonstrated excellent reliability. The interobserver ICCs ranged from 0.90 to 0.96, and the intraobserver ICCs ranged from 0.91 to 0.97, all indicating excellent agreement (ICC > 0.90) (Table 1).

**Table 1:** Inter-rater and test–retest reliabilities of quantitative MRI *T*2 mapping measurements in the lateral compartment

	Inter-rater reliability	Test–retest reliability
LFC superficial	0.96 (0.85–0.98)	0.97 (0.88–0.99)
LFC deep	0.93 (0.79–0.98)	0.92 (0.75–0.98)
LFC total	0.90 (0.70–0.97)	0.95 (0.83–0.98)
LTP superficial	0.94 (0.81–0.98)	0.90 (0.84–0.94)
LTP deep	0.96 (0.87–0.99)	0.91 (0.71–0.97)
LTP total	0.91 (0.71–0.97)	0.94 (0.79–0.98)

Values are presented as the intraclass correlation coefficient with the 95% confidence interval. LFC, lateral femoral condyle; LTP, lateral tibial plateau

### Patient demographics and preoperative characteristics

A total of 215 patients were screened for eligibility. Of these, 182 were excluded owing to the absence of either preoperative or postoperative *T*2 mapping MRI (*n* = 178) or incomplete clinical outcome data (*n* = 4). Consequently, 33 knees were included in the final analysis (13 in group I and 20 in group A). There were no significant between-group differences in age, sex distribution, height, weight, body mass index, preoperative Kellgren–Lawrence grade, or range of motion (Table 2).

**Table 2:** Comparison of patient demographic between group I and group A

	Group I	Group A	*p*-Value
Knees, number	13	20	
Sex, male/female	9/4	15/5	0.716
Age, years	48.2 ± 4.3	45.1 ± 8.9	0.524
Body mass index, kg/m^2^	28.6 ± 4.1	28.8 ± 4.2	0.758
Preoperative Kellgren–Lawrence grade, 0/1/2/3/4	0/1/5/7/0	0/5/9/5/1	0.365
Preoperative extension angle. °	2.9 ± 2.7	3.7 ± 5.0	0.861
Preoperative flexion angle. °	126.7 ± 10.6	120.8 ± 15.5	0.205

Values are presented as mean ± standard deviation or number. Group I: increased JLO/MPTA (JLO > 5° and/or MPTA > 94°); Group A: acceptable range JLO/MPTA (JLO < 5° and MPTA < 94°)

### Radiographic parameters

Preoperatively, group I had significantly lower HKA (172.7 ± 2.5° versus 174.9 ± 2.0°, *p* = 0.014), greater mLDFA (89.0 ± 1.2° versus 87.9 ± 1.8°, *p* = 0.030), and greater JLO (0.8 ± 2.2° versus −1.4 ± 1.7°, *p* = 0.018). Postoperatively, group I showed significantly greater mLDFA (*p* = 0.033), higher MPTA (*p* < 0.001), and greater JLO (*p* < 0.001), although there was no significant difference in HKA (*p* = 0.326). Patellar height indices (CDI, PTI) did not differ significantly between groups at either time point (Table 3). The correction angle was significantly greater in group I than in group A (9.3° ± 3.6° versus 6.6° ± 2.1°, *p* = 0.004).

**Table 3:** Comparison of radiographic variables between group I and group A

	Radiographic variables	Group I	Group A	*p*-Value
Preoperative	HKA, °	172.7 ± 2.5	174.9 ± 2.0	0.014^*^
	mLDFA, °	89.0 ± 1.2	87.9 ± 1.8	0.030^*^
	MPTA, °	85.2 ± 2.6	85.4 ± 1.7	0.785
	JLCA, °	3.7 ± 1.6	2.8 ± 2.1	0.110
	JLO, °	0.8 ± 2.2	−1.4 ± 1.7	]0.018^*^
	PTI	0.4 ± 0.1	0.4 ± 0.1	0.687
	CDI	1.0 ± 0.2	1.1 ± 0.2	0.893
Postoperative	HKA, °	182.7 ± 2.3	182.1 ± 1.5	0.326
	mLDFA, °	89.3 ± 1.4	87.9 ± 2.1	0.033^*^
	MPTA, °	94.5 ± 2.1	91.8 ± 1.0	< 0.001^*^
	JLCA, °	2.3 ± 2.1	2.0 ± 1.5	0.298
	JLO, °	6.2 ± 1.9	2.0 ± 1.3	< 0.001^*^
	CDI	1.0 ± 0.2	1.0 ± 0.2	0.924
	PTI	0.4 ± 0.1	0.4 ± 0.1	0.454

Values are presented as mean ± standard deviation. Group I: increased JLO/MPTA (JLO > 5° and/or MPTA > 94°); group A: acceptable range JLO/MPTA (JLO < 5° and MPTA < 94°). HKA, hip-knee-ankle angle; mLDFA, mechanical lateral distal femoral angle; MPTA, medial proximal tibial angle; JLCA, joint line congruence angle; JLO, joint line obliquity; CDI, Caton–Deschamps index; PTI, patellotrochlear index. *p < 0.05

### Quantitative MRI *T*2 mapping

#### Patellofemoral compartment

Preoperatively, no significant differences in *T*2 values were observed between group I and group A in any subregion or cartilage layer (*p* > 0.05). Similarly, no significant between-group differences were found postoperatively (*p* > 0.05, Table 4).

**Table 4:** Comparison of *T*2 values (ms) in the patellofemoral compartment between group I and group A

	Region of interest	Group I	Group A	*p*-Value
Preoperative *T*2 value	Patella superficial	54.6 ± 10.3	51.3 ± 11.6	0.392
	Patella deep	45.2 ± 9.5	41.1 ± 9.8	0.265
	Patella total	49.2 ± 8.6	47.0 ± 9.9	0.501
	Trochlea superficial	66.1 ± 10.3	65.0 ± 11.3	1.000
	Trochlea deep	53.4 ± 14.3	58.7 ± 12.1	0.316
	Trochlea total	60.3 ± 11.9	62.1 ± 10.3	0.548
Postoperative *T*2 value	Patella superficial	55.2 ± 11.8	53.2 ± 12.2	0.758
	Patella deep	46.9 ± 11.7	42.1 ± 9.9	0.207
	Patella total	50.6 ± 10.7	48.1 ± 10.0	0.501
	Trochlea superficial	65.8 ± 11.0	62.7 ± 10.3	0.548
	Trochlea deep	57.1 ± 12.7	58.9 ± 13.3	0.842
	Trochlea total	62.1 ± 11.1	61.0 ± 10.7	0.899
Δ*T*2 value (postoperative–preoperative)	Patella superficial	0.6 ± 7.1	1.9 ± 6.3	0.372
	Patella deep	1.7 ± 8.0	1.0 ± 5.1	0.624
	Patella total	1.4 ± 6.8	1.1 ± 4.9	1.000
	Trochlea superficial	− 0.3 ± 6.2	− 2.3 ± 6.0	0.413
	Trochlea deep	3.2 ± 9.3	0.1 ± 9.0	0.372
	Trochlea total	1.7 ± 5.1	− 1.1 ± 5.2	0.158

Values are presented as mean ± standard deviation. Group I: increased JLO/MPTA (JLO > 5° and/or MPTA > 94°); Group A: acceptable range JLO/MPTA (JLO < 5° and MPTA < 94°)

#### Lateral compartment

Preoperatively and at the postoperative assessment, no significant differences in *T*2 values were observed between groups in any lateral compartment cartilage layer (all *p* > 0.05; Table 5). However, Δ*T*2 values in the LTP were significantly greater in group I than in group A for the superficial (3.3 ± 6.5 ms versus −1.7 ± 5.9 ms, *p* = 0.022) and total (4.5 ± 5.7 ms versus −0.6 ± 5.0 ms, *p* = 0.012) layers.

**Table 5:** Comparison of *T*2 values (ms) in the lateral compartment between group I and group A

	Region of interest	Group I	Group A	*p*-Value
Preoperative *T*2 value	LFC superficial	58.7 ± 5.1	61.3 ± 9.7	0.235
	LFC deep	35.8 ± 14.1	39.6 ± 10.0	0.334
	LFC total	47.9 ± 8.7	50.1 ± 9.5	0.235
	LTP superficial	46.4 ± 6.7	46.6 ± 8.3	0.232
	LTP deep	23.8 ± 6.9	24.3 ± 9.3	0.420
	LTP total	34.3 ± 5.5	35.6 ± 8.6	0.649
Postoperative *T*2 value	LFC superficial	58.7 ± 5.1	59.4 ± 8.3	0.928
	LFC deep	40.3 ± 14.1	42.4 ± 9.3	0.758
	LFC total	48.5 ± 5.7	50.1 ± 8.5	0.434
	LTP superficial	49.8 ± 10.1	44.7 ± 7.3	0.080
	LTP deep	28.9 ± 6.4	26.3 ± 8.6	0.207
	LTP total	38.9 ± 7.8	34.9 ± 8.1	0.094
Δ*T*2 value (postoperative–preoperative)	LFC superficial	−0.0 ± 5.0	−1.8 ± 5.9	0.600
	LFC deep	4.5 ± 6.8	2.7 ± 8.6	0.309
	LFC total	0.6 ± 3.7	0.0 ± 5.5	0.834
	LTP superficial	3.3 ± 6.5	− 1.7 ± 5.9	0.022^*^
	LTP deep	5.1 ± 6.7	1.9 ± 6.2	0.158
	LTP total	4.5 ± 5.7	− 0.6 ± 5.0	0.012^*^

Values are presented as mean ± standard deviation. Group I: increased JLO/MPTA (JLO > 5° and/or MPTA > 94°); Group A: acceptable range JLO/MPTA (JLO < 5° and MPTA < 94°). LFC, lateral femoral condyle; LTP, lateral tibial plateau. *p < 0.05

In a sensitivity analysis adjusting for preoperative alignment differences (HKA, mLDFA, and JLO) using a general linear model with covariates, the between-group differences in Δ*T*2 for the lateral tibial plateau were no longer statistically significant (superficial layer: 3.2 ± 2.2 ms versus −1.7 ± 1.7 ms, *p* = 0.126; total layer: 3.3 ± 1.8 ms versus −0.2 ± 1.4 ms, *p* = 0.179).

### Clinical outcomes

Preoperative KOOS and WOMAC scores did not differ significantly between groups (all *p* > 0.05). At the final follow-up, group I had significantly worse KOOS–Symptoms (68.6 ± 13.6 versus 79.7 ± 11.0, *p* = 0.016), KOOS–QOL (48.5 ± 18.0 versus 62.3 ± 16.2, *p* = 0.022), and KOOS–Sport/Rec (52.7 ± 17.8 versus 66.0 ± 18.3, *p* = 0.040) compared with group A. No significant between-group differences were observed in KOOS–Pain, KOOS–ADL, or WOMAC subscales (all *p* > 0.05).

After adjusting for preoperative alignment parameters (HKA, mLDFA, and JLO), KOOS–Symptoms (64.6 ± 4.3 versus 81.2 ± 3.2, *p* = 0.016) and KOOS–QOL (45.0 ± 5.6 versus 64.6 ± 4.3, *p* = 0.020) remained significantly worse in group I. The magnitude of improvement in PROMs, expressed as ΔKOOS and ΔWOMAC, did not differ significantly between the two groups (all *p* > 0.05).

## Discussion

The most important finding of this study is that knees with increased joint line obliquity (JLO > 5°) and/or MPTA > 94° following MOWHTO showed greater increases in lateral compartment *T*2 values on quantitative MRI, suggesting early cartilage matrix changes, as well as worse postoperative patient-reported outcomes. However, as the between-group differences in Δ*T*2 values were no longer statistically significant after adjustment for preoperative alignment parameters that differed between groups, these findings should be interpreted with caution, and the influence of baseline alignment characteristics cannot be excluded. This is the first study to investigate cartilage changes after MOWHTO using quantitative MRI *T*2 mapping, allowing detection of early biochemical cartilage changes that are not identifiable on radiographs. Our results provide imaging-based evidence that supports previous biomechanical concerns regarding excessive valgus joint lines and suggest the importance of considering postoperative JLO during MOWHTO planning.

Radiographic progression of PF OA after MOWHTO has been frequently reported, although previous clinical studies have found no association with patient-reported outcomes [29, 30]. Biomechanical investigations have shown that MOWHTO can increase PF contact pressure in an angle-dependent manner [31, 32], with proximal tuberosity osteotomy markedly elevating PF pressure between 30° and 90° of flexion. In the present study, *T*2 values increased across all patellar regions in both groups; however, no significant differences were observed between groups I and A. The relatively small difference in correction angles in our cohort (mean 9.3° in group I, 6.6° in group A) compared with previous studies (> 10°) may have reduced the detectability of angle-dependent PF loading changes. Moreover, no significant between-group differences were found in patellar height—another factor associated with PFOA—indicating that PF cartilage changes cannot be attributed solely to variations in JLO or MPTA. Further research is needed to clarify the multifactorial determinants of PF OA progression after MOWHTO.

Few studies have investigated early cartilage changes in the lateral compartment following MOWHTO. Kumagai et al. reported significant deterioration of the ICRS grade in the LFC and LTP at second-look arthroscopy [33]. Furthermore, they found that lateral compartment cartilage degeneration was the only factor significantly associated with deterioration in both knee and function scores. In contrast, Kim et al. found that although patients with increased postoperative JLO more frequently experienced lateral-sided knee pain, there was no significant difference in arthroscopic cartilage deterioration in the lateral compartment between groups [8]. These findings indicate that the effect of lateral compartment cartilage degeneration on clinical outcomes remains controversial. In the present study, although Δ*T*2 values in the lateral tibial plateau were significantly greater in group I in the unadjusted analysis, this difference was no longer statistically significant after adjustment for preoperative alignment parameters that differed between groups. This suggests that baseline alignment characteristics may have contributed to the observed cartilage changes. In addition, cartilage changes may reflect the combined influence of preoperative bony morphology and postoperative alignment rather than the effect of postoperative JLO alone. Furthermore, correlation analyses between Δ*T*2 values in the lateral tibial plateau and postoperative PROMs or ΔPROMs did not reveal significant associations. Although lateral tibial plateau cartilage deterioration observed in group I could potentially contribute to future progression of lateral compartment osteoarthritis and worsening symptoms, this relationship could not be confirmed in the present study, possibly owing to the short follow-up period and limited sample size. Further studies with larger cohorts are warranted to clarify this association.

Postoperative increases in JLO after MOWHTO are known to result from multiple factors. In the present study, after adjusting for preoperative variables that differed significantly between groups (greater varus alignment, mLDFA, and JLO), the change in LTP *T*2 values was no longer statistically significant. This suggests that cartilage alterations in the lateral tibial plateau cannot be explained solely by postoperative increases in JLO or MPTA. In particular, the greater femoral varus (increased LDFA) and valgus JLO observed preoperatively in group I may have contributed to postoperative increases in JLO. This, in turn, could have led to cartilage deterioration in the LTP. Previous studies have shown that greater LDFA combined with increased JLCA markedly raises the risk of postoperative JLO > 5° [34] and that JLCA > 5° with preoperative JLO > 3° results in an almost 80% incidence of postoperative MPTA > 95° [7]. These findings highlight the importance of considering preoperative bone morphology, such as femoral varus morphology reflected by greater mLDFA, and a valgus JLO when planning knee osteotomy procedures.

Previous studies have reported mixed results regarding the influence of increased postoperative alignment parameters, such as MPTA and JLO, on clinical outcomes after MOWHTO. Akamatsu et al. found that knees with MPTA > 95° had significantly worse KOOS sports and recreation scores, suggesting that excessive correction may impair certain aspects of function [10]. In contrast, Goshima et al. reported no significant differences in KOOS or other major clinical scores between knees with MPTA > 95° and < 95°, indicating a minimal clinical impact [29]. In the present study, knees with increased JLO or MPTA showed inferior patient-reported outcomes at short-term follow-up. Notably, although absolute postoperative PROMs were inferior in the increased JLO/MPTA group, no significant differences were observed in ΔPROMs, suggesting that postoperative alignment may influence absolute postoperative outcomes rather than the magnitude of postoperative improvement. This finding suggests that the consideration of additional procedures to restore joint line horizontality, such as concomitant femoral osteotomy, may be warranted when excessive JLO is anticipated.

This study has several limitations. First, the sample size was relatively small, partly because quantitative *T*2 mapping MRI was available only at a specific imaging center. In addition, the specific MRI protocol used for *T*2 mapping in this study has since been discontinued and is no longer available in current clinical practice, which may limit the generalizability and reproducibility of our findings. The small sample size may have increased the risk of type II error, particularly for the negative findings in the patellofemoral compartment, and resulted in the exclusion of a substantial proportion of the screened cohort, introducing potential selection bias. However, a detailed comparison between included and excluded patients was not feasible owing to limited availability of baseline data for excluded cases. To address concerns regarding statistical power, a sensitivity analysis of the prespecified primary outcome (Δ*T*2 in the LTP total layer) suggested that the current sample size was sufficient to detect effects of this magnitude, whereas smaller effects—particularly in secondary outcomes—may have been underpowered. Second, the follow-up period was limited to 12 months postoperatively, potentially underestimating the long-term consequences of increased JLO/MPTA. Third, cartilage assessment was limited to the lateral and patellofemoral compartments because accurate segmentation of the medial compartment was not feasible in many patients owing to advanced chondral defects, and analyses relied on a single representative sagittal slice per compartment, which may not fully capture regional cartilage heterogeneity. Fourth, postoperative JLO and MPTA were combined for group classification based on consensus recommendations, precluding separate evaluation of their independent effects. Fifth, baseline differences in preoperative alignment parameters between groups may have influenced postoperative JLO; although statistical adjustment was performed, a matched study design (e.g., propensity score matching) would provide a more robust assessment. Finally, the use of different alignment guidance systems may represent a potential source of methodological bias, although no differences in correction accuracy or postoperative alignment were observed.

Despite these limitations, this is the first study to demonstrate both adverse effects on lateral tibial plateau cartilage health and worse clinical outcomes. Further research with larger cohorts and longer follow-up is warranted to confirm these findings and clarify the relationship between cartilage health and clinical outcomes.

## Conclusions

Despite similar overall limb alignment, patients with increased postoperative JLO and MPTA exhibited early degenerative changes in the lateral tibial cartilage and inferior patient-reported outcomes. These findings emphasize the importance of maintaining JLO within the acceptable range during MOWHTO to help preserve lateral compartment cartilage health, and improve longer term outcomes.

## Data Availability

The datasets generated and/or analyzed during the current study are available from the corresponding author on reasonable request.
